# Effect of mild roasting on Arabica and Robusta coffee beans contamination with polycyclic aromatic hydrocarbons

**DOI:** 10.1007/s13197-018-3532-0

**Published:** 2018-12-24

**Authors:** Marta Ciecierska, Dorota Derewiaka, Jolanta Kowalska, Ewa Majewska, Beata Drużyńska, Rafał Wołosiak

**Affiliations:** Division of Food Quality Evaluation, Faculty of Food Sciences, Warsaw University of Life Sciences, Nowoursynowska 159, 02-787 Warsaw, Poland

**Keywords:** PAHs, Coffee, Electric roasting, HPLC-FLD/DAD, GC/MS

## Abstract

The aim of this research was to establish the effect of mild roasting on coffee beans contamination level by polycyclic aromatic hydrocarbons (PAHs). The materials investigated were green Arabica and Robusta coffee beans imported from different countries, as well as those already roasted. The experiment was carried out in a coffee-roasting plant, with the use of an electric coffee roaster, at the temperature of 125–135 °C for 25–26 min. PAHs analysis was conducted by means of high-performance liquid chromatography with fluorescence and diode array detectors (HPLC-FLD/DAD). Results had been verified by means of gas chromatography with mass spectrometry. Contamination level for 19 PAHs, 15 of which were heavy PAHs included on the list of European Union Scientific Committee in Food, varied from 4.29 to 16.17 µg/kg in roasted coffee beans, whereas in green coffee beans varied from 8.66 to 76.63 µg/kg. The results of statistical analysis showed that the contamination level in roasted coffee beans was significantly lower than that in green beans. The applied parameters of roasting did not lead to the occurrence of conditions in which PAHs, especially heavy ones, would possibly be formed. On the contrary, the roasting process itself had significantly reduced the PAHs content in the final product. The reason for this phenomenon was relatively high volatility of light PAHs.

## Introduction

Polycyclic aromatic hydrocarbons (PAHs) are toxic compounds of various structure existing commonly in the natural environment. They originate from the incomplete combustion of organic matter. PAHs can be divided into light and heavy, depending on the number of aromatic rings in the molecule. Heavy PAHs are considerably more toxic than light ones (EFSA 2008; Murkovic et al. 2018; Singh et al. 2016).

In 2002, the European Commission’s Scientific Committee on Food (SCF) ruled that 15 PAHs are genotoxic carcinogens (SCF 2002). As stated in the European Union Commission Recommendation from 4 February 2005, further scientific investigation of these PAHs content in foodstuffs is required (Commission of the European Communities 2005). Subsequently, in 2008 European Food Safety Authority (EFSA) stated that 4 heavy, specific marker PAHs (benzo[a]pyrene (B[a]P), benzo[a]anthracene (B[a]A), benzo[b]fluoranthene (B[b]F) and chrysene (Chr)) are the most suitable indicators of foodstuffs PAHs content, and therefore their presence in food should be monitored. The same was stated in Commission Regulation (EU) No. 835/2011 (Commission of the European Communities 2011a; EFSA 2008).

PAHs have been detected in soil, sediments, water and air (Hong et al. 2016; Tomaz et al. 2017), as well as in foodstuffs (Ciecierska and Obiedziński 2013b; Murkovic et al. 2018; Singh et al. 2016; Surma et al. 2018). Food contamination with PAHs may both be a result of general environment pollution, heat processing during food production and its preparation for consumption. Especially roasting, smoking, grilling and direct drying favour PAHs formation (Chung et al. 2011; SCF 2002; Singh et al. 2016). The PAHs contamination level in a given product depends on many factors, such as this product's distance from heat source, fuel used for heating, level of product processing, cooking duration (Singh et al. 2016).

Foodstuffs with high fat content are especially vulnerable to PAHs contamination. The reason for this is fat being a carrier of hydrophobic PAHs. Coffee beans usually contain from 10 to 17% of fat and may become environmentally contaminated with PAHs. However, in this case, drying and roasting process is probably the main source of PAHs contamination (Guatemala-Morales et al. 2016; Houessou et al. 2008; Pissinatti et al. 2015). Coffee roasting conditions, such as time and temperature, are to be controlled so that PAHs formation may be reduced or at least minimised. Therefore, the above-mentioned conditions are crucial factors in PAHs formation (Guatemala-Morales et al. 2016; Houessou et al. 2007; Stanciu et al. 2008).

The chemical contaminants content levels in food, including PAHs levels, should be consistent with the ALARA ("As Low As Reasonably Achievable") rule. Since natural gas-powered roasters emitting relatively high temperature are generally used in coffee roasting on a mass scale, the use of electric coffee roaster, emitting unusually low roasting temperature may prove beneficial. Unfortunately, the majority of publications dealing with PAHs occurrence in coffee do not describe the effect of mild roasting on coffee contamination with above-mentioned substances. Therefore, the main aim of this research was to assess the effect of mild roasting conditions on coffee beans PAHs contamination level. The study included PAHs determination with the use of HPLC-FLD/DAD method. Arabica and Robusta coffee beans of different origins and different state: both green and roasted in an electric coffee roaster were examined. During previous studies concerning PAHs occurrence in food, it was established that 4 light PAHs, known as phenanthrene (Phen), anthracene (Anthr), fluoranthene (F) and pyrene (Pyr), had always been considered dominant in PAHs qualitative profiles (Ciecierska and Obiedziński 2010; Murkovic et al. 2018). Therefore, apart from 15 heavy PAHs, which were listed to be monitored by the SCF, also the above-mentioned 4 light PAHs listed by the United States Environmental Protection Agency (US EPA) were determined in analysed coffee samples. Particular attention was also paid to 4 heavy and marker PAHs content evaluation.

## Material and methods

### Samples

The materials investigated were green beans of *Coffea Arabica* L. and *Coffea canephora* (“*Coffea robusta*”) imported from different countries, as well as their roasted equivalents. The countries of import were, in case of Arabica coffee beans, Brazil, Colombia, Cuba, Ethiopia, Indonesia, Kenya, Peru and Tanzania, and in case of Robusta coffee beans—Cameroon, India, Indonesia, Ivory Coast, Thailand, Uganda, Vietnam and Zaire. The analysed coffee brands intentionally were not revealed. Nine samples of every coffee beans (including three various coffee batches) were studied.

### Coffee roasting

Green coffee beans (three 2 kg batches) had been roasted for 25–26 min. at the temperature ranging from 125 to 135 °C with the use of an electric coffee roaster, brand Toper Electric TKMSX-3-E (Halifax, United Kingdom). The process took place in one of Warsaw coffee-roasting plants. The conditions used for the research had been tested for some time in the above-mentioned coffee-roasting plant. Coffee roasted in the way mentioned turned out to be appreciated by consumers, who would praise it for its smell and taste.

### Chemicals and materials

Solvents of an analytical grade for residue analysis (HPLC gradient grade) used in the research: cyclohexane, ethyl acetate, hexane, acetone, and acetonitrile, as well as anhydrous sodium sulphate (> 99.0% of analytical purity), had been purchased from POCH (Gliwice, Poland). Deionized water had been obtained from a water purification device (Millipore Milli-Q). Polytetrafluoroethylene (PTFE) philtres (25 mm i.d., 1 µm pore size) had been provided by Bio Analytic company (Gdańsk, Poland).

Two standard PAHs mixtures: PAH-Mix 183 and PAH-Mix 9 (Dr Ehrenstorfer, 10 ng/µl, in acetonitrile) had been provided by Witko company (Łódź, Poland). PAH-Mix 183 includes 15 compounds listed by the SCF: cyclopenta[c,d]pyrene (C[cd]P), B[a]A, Chr, 5-metylchrysene (5-MChr), benzo[j]fluoranthene (B[j]F), B[b]F, benzo[k]fluoranthene (B[k]F), B[a]P, dibenzo[a,h]anthracene (D[ah]A), benzo[g,h,i]perylene (B[ghi]P), indeno[c,d]pyrene (I[cd]P), dibenzo[a,l]pyrene (D[al]P), dibenzo[a,e]pyrene (D[ae]P), dibenzo[a,i]pyrene (D[ai]P) and dibenzo[a,h]pyrene (D[ah]P). PAH-Mix 9 consists of 16 PAHs from the US EPA list. This particular mixture had been used only for the analysis of 4 light PAHs: Phen, Anthr, F and Pyr.

### Extraction and clean-up

Extraction, as well as purification procedure, was carried out with the use of the method described by Ciecierska and Obiedziński (2013a), although some modifications to the sample weight and an additional purification step, in which silica gel column was used, were added. To sum up with, homogenised and ground 7.5 g sample of coffee beans, dried with anhydrous sodium sulphate, was placed in an Erlenmeyer flask with 50 ml of hexane/acetone (60:40, v/v) into an ultrasonic bath for 30 min. The solution was then filtered and evaporated in a rotary vacuum evaporator (Büchi No. 141,397 brand, Flawil, Switzerland). Subsequently, evaporated solution was dissolved in 0.5 ml of cyclohexane, subjected to clean-up on silica gel column and eluted by cyclohexane. The first 10 ml of an eluate was discarded and subsequent 75 ml was collected, concentrated and dissolved in 5 ml of cyclohexane/ethyl acetate (50:50, v/v). The obtained extract was then filtered with the use of PTFE philtre.

A purification procedure also described by Ciecierska and Obiedziński (2013a) was carried out in order to isolate PAHs. Size exclusion chromatography (SEC) on TSK Gel G1000HXL column (300 × 7.8 mm, 5 μm, Polygen company, Gliwice, Poland) was applied. The extract purified in this way was dissolved in acetonitrile after solvent evaporation. Subsequently, it underwent chromatographic analysis.

### HPLC-FLD/DAD analysis

PAHs determination was carried out with the use of Shimadzu HPLC system, that is with the use of liquid chromatograph LC-10ATVP and selective detectors: fluorescence detector RF-10AXL and diode array detector SPD-M10AVP. Column BAKERBOND PAH-16 Plus (Baker brand, 250 × 3 mm, 5 µm) supplied by Witko company (Łódź, Poland) was used for PAHs separations. Details of the chromatographic analysis together with the analysed compounds detection conditions had been described by Ciecierska and Obiedziński (2013a). Briefly, a gradient method, in which 0.5 ml/min flow rate of acetonitrile/water (50:50) and acetonitrile, was used for elution. The following fluorescence detection parameters were applied in order to detect particular PAHs: 256/370 nm, 270/420 nm, 270/500 nm, 270/470 nm (excitation/ emission wavelengths). The diode array detector working at 254 nm was used to detect only one compound—C[cd]P, which does not fluoresce in a natural way.

### Quantification and validation of method

An external standard method with the use of two previously described PAHs mixtures (PAH-Mix 183 and PAH-Mix 9, Dr Ehrenstorfer brand) was applied to qualitative and quantitative PAHs analysis. Six standard solutions of different concentration (from 1 to 50 µg/l) were prepared on the basis of the above-mentioned standard mixtures in order to calibrate and validate the method. The calibration curves for all 19 PAHs, their correlation coefficients (r^2^), as well as method linearity range, are shown in Table 1. The linearity of the method was proven for almost all compounds within the range of analysed concentrations.

**Table 1:** The HPLC-FLD/DAD method performance for the PAHs analysis in roasted Arabica coffee (Brazil)

PAH	Linearity range (µg/l)	Calibration curve^a^	Correlation coefficient r^2^	LOD (µg/kg)	LOQ (µg/kg)	Recovery^b^ (%)	RSD^b^ (%)	HORRAT_R_ value^b^
Phen	1–50	y = 149,144x + 31,879	0.9998	0.06	0.11	71.9	9.5	0.8
Anthr	1–50	y = 117,707x + 4272.1	0.9999	0.07	0.14	73.0	9.0	0.7
F	1–50	y = 18,392x − 2547	0.9999	0.13	0.26	85.0	8.8	0.7
Pyr	1–50	y = 88,196x + 1193.6	0.9998	0.08	0.16	86.5	8.9	0.7
C[cd]P	2–50	y = 122.78x + 14.9	0.9993	0.47	0.94	110.0	7.9	0.7
B[a]A	1–50	y = 159,530x − 45,297	0.9997	0.05	0.10	86.4	5.1	0.4
Chr	1–50	y = 51,379x − 13,012	0.9996	0.08	0.16	84.5	5.4	0.4
5-MChr	1–50	y = 97,566x − 6394.4	0.9999	0.07	0.15	84.6	6.3	0.5
B[j]F	2–50	y = 1600.7x + 196.91	0.9997	0.32	0.64	82.4	6.7	0.6
B[b]F	1–50	y = 78,977x − 22,630	0.9998	0.10	0.20	80.1	4.6	0.4
B[k]F	1–50	y = 78,142x − 4724.2	0.9997	0.10	0.19	81.3	5.8	0.5
B[a]P	1–50	y = 35,742x − 8979.4	0.9999	0.12	0.24	83.5	6.0	0.5
D[ah]A	1–50	y = 39,684x − 8533	0.9997	0.13	0.26	77.0	6.7	0.6
D[al]P	2–50	y = 359.96x − 154.23	0.9995	0.30	0.60	77.4	7.7	0.6
B[ghi]P	1–50	y = 14,600x − 5088.6	0.9997	0.15	0.30	85.8	7.0	0.6
I[cd]P	1–50	y = 12,337x − 839.5	0.9997	0.28	0.56	81.9	7.6	0.6
D[ae]P	1–50	y = 8144.2x + 456.41	0.9997	0.29	0.59	83.1	7.3	0.6
D[ai]P	1–50	y = 226,619x − 96,869	0.9997	0.13	0.25	85.5	7.2	0.6
D[ah]P	1–50	y = 172,110x − 68,308	0.9997	0.16	0.33	76.6	7.9	0.7

^a^y—peak area, x—concentration (µg/l);

^b^Mean values of recovery, RSD and HORRAT_R_ of three different levels of sample fortification

Both the limit of detection check and the limit of quantification check (LOD and LOQ check), as well as recovery experiments, were conducted in order to validate the method. LOD and LOQ for all analysed PAHs are shown in Table 1. For recovery experiments, samples of roasted Arabica coffee from Brazil were fortified (spiked) at three concentration levels of PAHs standard mixtures, and these levels were 1, 10 and 50 µg/kg. All samples, both those fortified and those unfortified, were analysed thrice. The values of the mean recovery, as well as the relative standard deviation (RSD) and the HORRAT_R_ values (used as a measure of the method precision) for three-level fortification, all calculated in accordance with Commission Regulation (EU) No. 836/2011 (Commission of the European Communities 2011b), are also shown in Table 1.

### Results confirmation by GC/MS

The results obtained by HPLC-FLD/DAD method were confirmed with the use of gas chromatography with mass spectrometry (GC/MS) in accordance with the method described by Ciecierska and Obiedziński (2013a).

### Statistical analysis

Statistica 10.0 programme was used to carry out statistical analysis of the data obtained. The significance of the differences in the mean content of 19 PAHs, 4 light and 4 heavy and marker PAHs, between green and roasted coffee beans, was proven by the use of multiple comparisons analysis and Tukey’s test, at significance level α = 0.05.

## Results and discussion

**Fig. 1:**
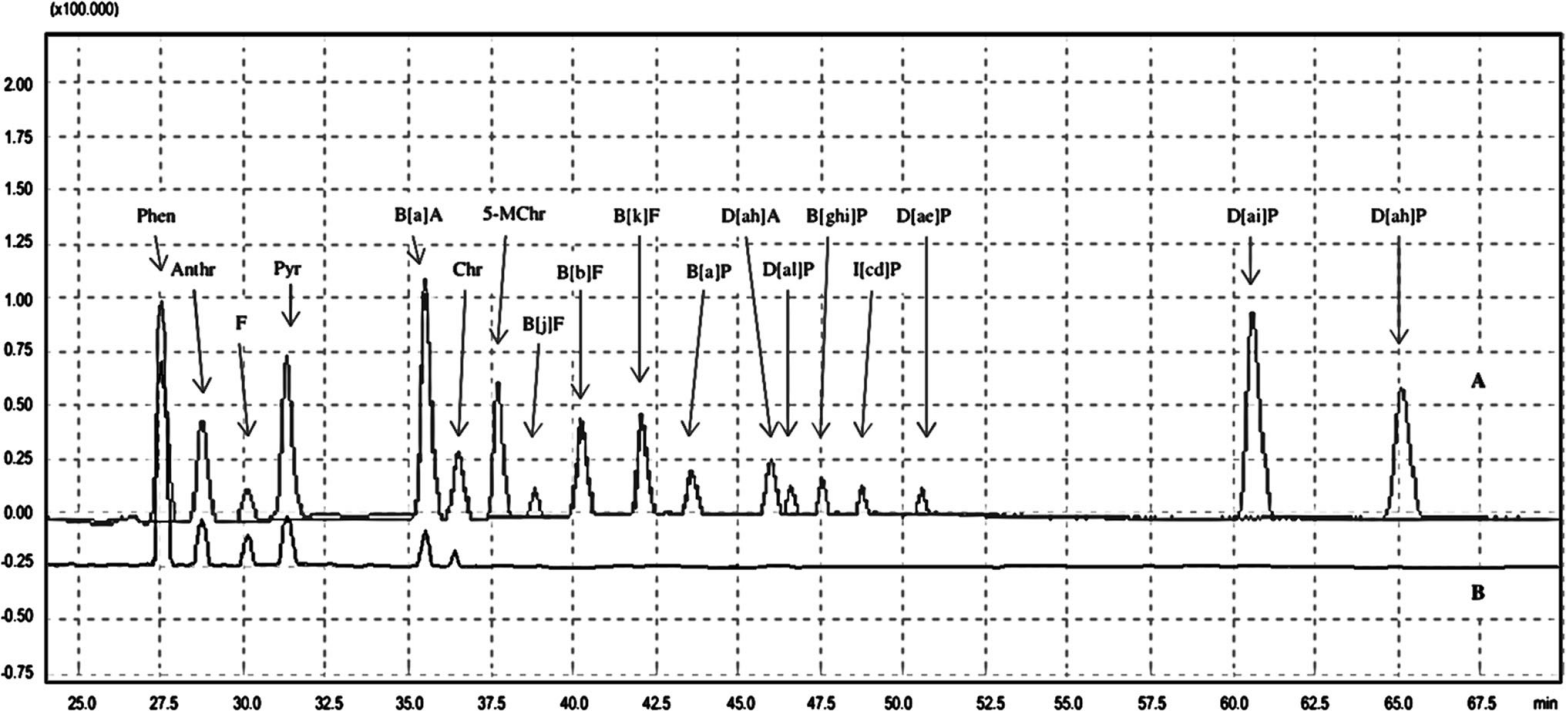
Chromatograms of the analysed PAHs in: **a** 15 PAHs listed by SCF (PAH-Mix 183, Dr. Ehrenstorfer, 10 pg/µl) and 16 US EPA PAHs (PAH-Mix 9, Dr. Ehrenstorfer, 10 pg/µl) and **b** the roasted Arabica coffee (Brazil)

The performance parameters obtained (including LOD, LOQ, HORRAT_R_ values and recovery) showed that HPLC-FLD/DAD method meets requirements of Commission Regulation (EU) No. 836/2011 referring to the methods of 4 marker PAHs analysis in food. Also, for the remaining compounds, such as 4 light PAHs listed by EPA and remaining PAHs from the SCF list, satisfactory results of the method performance were stated (Table 1). The chromatograms of the 15 analysed PAHs appearing on the SCF list and 16 PAHs described by US EPA, as well as one of roasted Arabica coffee, are shown in Fig. 1.

Results of the mean PAHs content examination, regarding the content of both particular compounds and a sum of the 19 PAHs (of which 4 light, 4 heavy and marker PAHs) in examined green Arabica and Robusta coffee beans of different place of origin and their roasted equivalents are shown in Tables 2 and 3. After having compared the results from HPLC-FLD/DAD method and GC/MS method, the researchers noticed that the differences between these two methods are not statistically significant for all products which had undergone examination.

**Table 2:** Mean content of PAHs in green and roasted Arabica coffees derived from different countries (μg/kg)

PAH	Brazil		Colombia		Cuba		Ethiopia	
	Green	Roasted	Green	Roasted	Green	Roasted	Green	Roasted
Phen	4.99 ± 0.62	1.57 ± 0.24	19.45 ± 1.93	4.38 ± 0.43	4.73 ± 0.55	1.33 ± 0.20	31.96 ± 3.41	2.37 ± 0.35
Anthr	0.80 ± 0.05	0.55 ± 0.03	2.11 ± 0.25	0.75 ± 0.09	0.95 ± 0.07	0.61 ± 0.02	1.25 ± 0.20	0.46 ± 0.05
F	1.50 ± 0.22	1.35 ± 0.18	3.46 ± 0.41	2.05 ± 0.23	1.32 ± 0.16	1.02 ± 0.11	6.40 ± 0.65	1.25 ± 0.10
Pyr	1.23 ± 0.13	1.13 ± 0.11	3.17 ± 0.31	2.22 ± 0.24	1.17 ± 0.19	0.99 ± 0.08	4.74 ± 0.49	1.01 ± 0.08
B[a]A	0.28 ± 0.05	0.21 ± 0.05	0.35 ± 0.05	0.25 ± 0.04	0.26 ± 0.03	0.18 ± 0.06	0.25 ± 0.10	0.14 ± 0.07
Chr	0.26 ± 0.04	0.17 ± 0.06	0.39 ± 0.06	0.27 ± 0.06	0.23 ± 0.07	0.16 ± 0.05	0.32 ± 0.12	0.18 ± 0.10
5-MChr	nd^a^	nd	nd	nd	nd	nd	nd	nd
B[b]F	nd	nd	nd	nd	nd	nd	0.14 ± 0.03	nd
B[k]F	nd	nd	nd	nd	nd	nd	nd	nd
Σ 19 PAHs^b^	9.06 ± 0.97^a1^	4.98 ± 0.51^b1^	28.93 ± 2.91^a2^	9.92 ± 0.99^b2^	8.66 ± 0.88^a3^	4.29 ± 0.46^b3^	45.06 ± 4.94^a4^	5.41 ± 0.63^b4^
Σ 4 light PAHs (Phen, Anthr, F, Pyr)	8.52 ± 0.93^a9^	4.60 ± 0.47^b9^	28.19 ± 2.90^a10^	9.40 ± 0.97^b10^	8.17 ± 0.82^a11^	3.95 ± 0.41^b11^	44.35 ± 4.75^a12^	5.09 ± 0.58^b12^
Σ 4 heavy PAHs (B[a]A, Chr, B[b]F, B[a]P)	0.54 ± 0.08^a17^	0.38 ± 0.11^a17^	0.74 ± 0.11^a18^	0.52 ± 0.10^a18^	0.49 ± 0.09^a19^	0.34 ± 0.11^a19^	0.71 ± 0.20^a20^	0.32 ± 0.16^a20^

PAH	Indonesia		Kenya		Peru		Tanzania	
	Green	Roasted	Green	Roasted	Green	Roasted	Green	Roasted
Phen	28.37 ± 3.21	1.95 ± 0.20	34.55 ± 4.03	3.32 ± 0.41	23.40 ± 3.15	5.50 ± 0.66	31.77 ± 3.55	3.58 ± 0.65
Anthr	1.25 ± 0.20	0.16 ± 0.02	4.56 ± 0.15	1.44 ± 0.16	1.87 ± 0.23	0.66 ± 0.08	4.80 ± 0.46	1.27 ± 0.10
F	10.85 ± 1.09	1.10 ± 0.17	11.45 ± 1.01	1.63 ± 0.25	4.40 ± 0.53	3.67 ± 0.35	14.54 ± 1.42	1.55 ± 0.11
Pyr	4.95 ± 0.65	0.99 ± 0.10	5.97 ± 0.80	1.10 ± 0.16	3.35 ± 0.35	2.73 ± 0.32	6.65 ± 0.54	0.99 ± 0.09
B[a]A	0.50 ± 0.04	0.35 ± 0.11	0.41 ± 0.09	0.30 ± 0.08	0.33 ± 0.04	0.23 ± 0.05	0.66 ± 0.15	0.34 ± 0.04
Chr	0.61 ± 0.16	0.51 ± 0.06	0.52 ± 0.11	0.38 ± 0.09	0.55 ± 0.07	0.41 ± 0.04	0.74 ± 0.18	0.32 ± 0.03
5-MChr	0.25 ± 0.06	0.15 ± 0.02	nd	nd	nd	nd	0.19 ± 0.04	nd
B[b]F	0.29 ± 0.08	0.17 ± 0.04	0.19 ± 0.05	nd	nd	nd	0.25 ± 0.04	nd
B[k]F	0.25 ± 0.05	0.14 ± 0.03	nd	nd	nd	nd	nd	nd
Σ 19 PAHs^b^	47.32 ± 5.55^a5^	5.57 ± 0.69^b5^	57.65 ± 6.10^a6^	8.17 ± 0.99^b6^	33.90 ± 4.10^a7^	13.20 ± 1.49^b7^	59.60 ± 6.18^a8^	8.05 ± 0.97^b8^
Σ 4 light PAHs (Phen, Anthr, F, Pyr)	45.42 ± 5.15^a13^	4.25 ± 0.47^b13^	56.53 ± 5.98^a14^	7.49 ± 0.84^b14^	33.02 ± 4.01^a15^	12.56 ± 1.41^b15^	57.76 ± 5.97^a16^	7.39 ± 0.95^b16^
Σ 4 heavy PAHs (B[a]A, Chr, B[b]F, B[a]P)	1.40 ± 0.29 ^a20^	1.03 ± 0.21^a21^	1.12 ± 0.24^a21^	0.68 ± 0.16^a22^	0.88 ± 0.11^a22^	0.64 ± 0.09^a23^	1.65 ± 0.35^a24^	0.66 ± 0.07^b24^

C[cd]P, B[j]F, B[a]P, D[ah]A, D[al]P, B[ghi]P, I[cd]P, D[ae]P, D[ai]P, D[ah]P were not detected in any of analysed samples

^a^*nd* not detected

^b^Different letters by the same number (meaning one comparison) below the mean values of sum of 19 PAHs, 4 light PAHs or 4 heavy PAHs indicate statistically significant difference between means at α = 0.05 level;

**Table 3:** Mean content of PAHs in green and roasted Robusta coffees derived from different countries (μg/kg)

PAH	Cameroon		India		Indonesia		Ivory Coast	
	Green	Roasted	Green	Roasted	Green	Roasted	Green	Roasted
Phen	30.33 ± 3.41	4.44 ± 0.64	14.99 ± 2.30	9.43 ± 0.93	30.20 ± 3.25	3.60 ± 0.33	32.55 ± 3.52	11.45 ± 1.20
Anthr	5.54 ± 0.54	1.43 ± 0.12	2.19 ± 0.24	1.16 ± 0.25	1.66 ± 0.35	0.27 ± 0.03	6.51 ± 0.55	0.89 ± 0.15
F	12.22 ± 1.25	1.64 ± 0.13	4.55 ± 0.42	2.53 ± 0.32	10.27 ± 1.01	2.59 ± 0.27	18.80 ± 1.99	1.67 ± 0.35
Pyr	8.10 ± 0.62	1.05 ± 0.09	2.97 ± 0.26	2.11 ± 0.27	5.58 ± 0.62	1.42 ± 0.23	17.33 ± 1.88	1.29 ± 0.34
B[a]A	0.50 ± 0.08	0.34 ± 0.06	0.39 ± 0.01	0.25 ± 0.05	0.32 ± 0.15	0.25 ± 0.10	0.40 ± 0.14	0.33 ± 0.14
Chr	0.75 ± 0.10	0.39 ± 0.08	0.37 ± 0.08	0.27 ± 0.06	0.26 ± 0.13	0.27 ± 0.09	0.52 ± 0.18	0.28 ± 0.08
5-MChr	0.25 ± 0.04	nd	nd	nd	0.23 ± 0.11	0.17 ± 0.04	0.22 ± 0.08	nd
B[b]F	0.17 ± 0.03	nd	nd	nd	nd	nd	0.30 ± 0.14	0.26 ± 0.10
Σ 19 PAHs^b^	58.36 ± 6.01^a1^	9.29 ± 1.06^b1^	25.46 ± 3.30^a2^	15.75 ± 1.87^b2^	48.52 ± 6.08^a3^	8.57 ± 0.95^b3^	76.63 ± 8.20^a4^	16.17 ± 2.14^b4^
Σ 4 light PAHs (Phen, Anthr,								
F, Pyr)	56.19 ± 5.80^a9^	8.56 ± 0.97^b9^	24.70 ± 3.22^a10^	15.23 ± 1.77^b10^	47.71 ± 5.19^a11^	7.88 ± 0.85^b11^	75.19 ± 7.74^a12^	15.30 ± 1.89^b12^
Σ 4 heavy PAHs								
(B[a]A, Chr, B[b]F, B[a]P)	1.42 ± 0.22^a17^	0.73 ± 0.14^b17^	0.76 ± 0.09^a18^	0.52 ± 0.11^a18^	0.58 ± 0.27^a19^	0.52 ± 0.17^a19^	1.22 ± 0.43^a20^	0.87 ± 0.22^a20^

PAH	Thailand		Uganda		Vietnam		Zaire	
	Green	Roasted	Green	Roasted	Green	Roasted	Green	Roasted
Phen	19.44 ± 1.95	5.89 ± 0.60	18.56 ± 2.50	7.44 ± 0.75	5.15 ± 0.58	2.93 ± 0.37	20.90 ± 2.30	6.22 ± 0.64
Anthr	2.13 ± 0.21	0.57 ± 0.05	0.85 ± 0.18	0.16 ± 0.03	0.52 ± 0.10	0.16 ± 0.02	1.94 ± 0.12	0.45 ± 0.06
F	5.88 ± 0.67	1.54 ± 0.21	5.44 ± 0.55	1.62 ± 0.26	1.80 ± 0.33	1.19 ± 0.22	6.38 ± 0.62	1.76 ± 0.20
Pyr	2.31 ± 0.32	1.02 ± 0.15	2.10 ± 0.28	1.33 ± 0.22	1.79 ± 0.35	1.17 ± 0.24	2.46 ± 0.23	1.21 ± 0.18
B[a]A	0.35 ± 0.06	0.27 ± 0.04	0.33 ± 0.06	0.25 ± 0.06	0.17 ± 0.02	0.15 ± 0.05	0.39 ± 0.07	0.22 ± 0.04
Chr	0.33 ± 0.08	0.25 ± 0.03	0.28 ± 0.03	0.23 ± 0.03	0.25 ± 0.04	0.17 ± 0.03	0.30 ± 0.05	0.26 ± 0.05
5-MChr	nd	nd	nd	nd	nd	nd	nd	nd
B[b]F	nd	nd	nd	nd	nd	nd	nd	nd
Σ 19 PAHs^b^	30.84 ± 3.28^a5^	9.54 ± 1.07^b5^	27.56 ± 3.54^a6^	11.03 ± 1.33^b6^	9.68 ± 1.07^a7^	5.77 ± 0.86^b7^	32.37 ± 3.38^a8^	10.12 ± 1.16^b8^
Σ 4 light PAHs (Phen, Anthr,								
F, Pyr)	30.16 ± 3.19^a13^	9.02 ± 1.01^b13^	26.95 ± 3.51^a14^	10.55 ± 1.24^b14^	9.26 ± 1.01^a15^	5.45 ± 0.83^b15^	31.68 ± 3.27^a16^	9.64 ± 1.08^b16^
Σ 4 heavy PAHs								
(B[a]A, Chr, B[b]F, B[a]P)	0.68 ± 0.12^a21^	0.52 ± 0.07^a21^	0.61 ± 0.09^a22^	0.48 ± 0.08^a22^	0.42 ± 0.06^a23^	0.32 ± 0.08^a23^	0.69 ± 0.12^a24^	0.48 ± 0.09^a24^

C[cd]P, B[j]F, B[k]F, B[a]P, D[ah]A, D[al]P, B[ghi]P, I[cd]P, D[ae]P, D[ai]P, D[ah]P were not detected in any of analysed samples

^a^*nd* not detected

^b^Different letters by the same number (meaning one comparison) below the mean values of sum of 19 PAHs, 4 light PAHs or 4 heavy PAHs indicate statistically significant difference between means at α = 0.05 level;

The qualitative profiles of PAHs in green coffee beans, as well as in their roasted equivalents, were similar. First of all, all the profiles showed relatively high content of 4 light PAHs. In green Arabica coffee beans, the light PAHs constituted from 94 to 98% of all analysed PAHs content, whereas in the roasted ones from 76 to 95%. In case of green Robusta coffee beans light PAHs constituted from 96 to 98% of all PAHs content, while in the roasted beans this value remained within the range of 92–97%. Two compounds from the group of 15 heavy PAHs, such as B[a]A and Chr, were detected in all examined coffee beans. Also 5-MChr, B[b]F and B[k]F were noticed in some samples. The rest of heavy PAHs (B[j]F, B[a]P, D[ah]A, B[ghi]P, I[cd]P and the most toxic dibenzopyrenes) were not detected both in green and in roasted coffee beans. It was therefore confirmed that heavy PAHs, genotoxic, mutagenic and carcinogenic properties of which have been stated, constituted low (from 2 to 24%) percentage of the total PAHs content in analysed coffee beans. Furthermore, in the majority of samples, 4 marker PAHs (B[a]P, B[a]A, B[b]F and Chr) constituted between 2 and 18% of all examined PAHs.

As the statistical analysis showed, significant differences in the total 19 PAHs content were stated between individual coffee beans originating from different countries, both for green and roasted coffee beans. In case of green Arabica coffee beans the highest level of PAHs contamination in the field of statistical significance was observed in coffee beans from Tanzania and Kenya—respectively 59.60 and 57.65 μg/kg. However, the lowest level of 19 PAHs content was detected in Arabica coffee beans originating from Cuba and Brazil. This PAHs contamination level was, respectively, 8.66 μg/kg and 9.06 μg/kg. Among roasted Arabica coffee beans the statistically significant, highest level of 19 PAHs contamination was detected in coffee beans from Peru (13.20 μg/kg), while the lowest contamination level was observed in coffee beans from Cuba (4.29 μg/kg).

In the group of green Robusta coffee beans, statistically the highest level of 19 PAHs contamination was determined in coffee from Ivory Coast (76.63 μg/kg). The lowest level of total PAHs content was noted in coffee beans from Vietnam (9.68 μg/kg). After roasting statistically the highest level of total PAHs contamination was observed in coffee beans from Ivory Coast (analogous to the one noticed in green coffee beans) and India (respectively 16.17 μg/kg and 15.75 μg/kg). Contrarily, the lowest 19 PAHs concentration level was detected in coffee from Vietnam—5.77 μg/kg (as in green coffee beans).

As far as the essential aim of this research is concerned, the results obtained confirmed the influence of mild roasting on the PAHs contamination level in analysed coffee beans. In case of both Arabica and Robusta coffee, lower levels of total PAHs content in the field of statistical significance were detected in roasted coffee beans, not their raw equivalents.

As a result of the applied roasting process, even more than sevenfold and eightfold decrease in the 19 PAHs content level was observed in Arabica coffee beans from Indonesia (from 47.32 to 5.57 μg/kg), Ethiopia (from 45.06 to 5.41 μg/kg), Tanzania (from 59.60 to 8.05 μg/kg) and Kenya (from 57.65 to 8.17 μg/kg). However, the lowest but still statistically significant decrease in 19 PAHs content (about twice and thrice) was noticed in coffee beans from Brazil (from 9.06 to 4.98 μg/kg), Cuba (from 8.66 to 4.29 μg/kg), Colombia (from 28.93 to 9.92 μg/kg) and Peru (from 33.90 to 13.20 μg/kg).

After the roasting of Robusta coffee beans, about sixfold decrease in the total 19 PAHs content was observed in coffee beans from Cameroon (from 58.36 to 9.29 μg/kg) and Indonesia (from 48.52 to 8.57 μg/kg). For other roasted Robusta coffee beans, the total PAHs contamination level was reduced from fivefold to 1.6-fold in comparison with their green equivalents.

It was also statistically confirmed that the decrease of the total 19 PAHs content on dry matter of the coffee beans as a consequence of the roasting is even more statistically significant than that stated above (for the product). Furthermore, after the roasting statistically significant, 4 light PAHs content reduction was also confirmed for every sample analysed. The reason for this research result may be relatively high volatility of light PAHs occurring during the roasting process applied. However, after the roasting process, reduction of the sum of 4 marker and heavy PAHs was not statistically significant in the vast majority of samples (88%). Moreover, the level of 4 marker and heavy PAHs contamination of analysed Arabica and Robusta coffee beans was relatively low especially in the roasted coffee beans, where it varied from 0.32 to 1.03 µg/kg. The level of 4 heavy PAHs content in raw coffee beans was in the range of 0.42–1.65 µg/kg.

According to other researchers, the B[a]P content for roasted coffee beans remained within the range of 0.10–0.51 μg/kg and the total PAHs concentration level varied from 1.00 to 32.52 μg/kg (Houessou et al. 2007; Lee and Shin 2010; Pissinatti et al. 2015; Stanciu et al. 2008). Other scientific reports showed that the B[a]P content in ground and instant coffee ranges from less than 0.01–1.2 μg/kg, whereas in the heavily roasted coffee beans the substance concentration level reaches 22.7 μg/kg (Houessou et al. 2006; Lai et al. 2004). In another work, concerning PAHs content in coffee roasted in a spouted bed reactor, the B[a]P concentration level could not be quantified, although the level for 16 EPA PAHs ranged from 3.5 to 16.4 μg/kg (Guatemala-Morales et al. 2016). In this research B[a]P was not detected in any of the analysed samples of both green and roasted coffee beans. As far as the level of 4 heavy PAHs regulated by the European Union is concerned, in the survey performed in Brazil the level for these compounds turned out to remain in the range from 1.00 to 3.98 µg/kg (Pissinatti et al. 2015).

Other studies, in which roasted coffee PAHs contamination was examined, showed that light PAHs, such as Phen, Anthr, F and Pyr, are always predominant in the PAHs contamination profile (Guatemala-Morales et al. 2016; Houessou et al. 2006). Therefore, qualitative and quantitative PAHs profiles of analysed coffee samples, presented in this paper, are similar to the results of above-mentioned works.

The obtained results showed considerable contamination levels' variety in individual products, both in the group of green and roasted coffee. PAHs contamination level in agricultural products mainly depends on the place of origin and its industrialization level. A wide range of PAHs emission sources, as well as vehicular traffic, affects the latter (Ciecierska and Obiedziński 2013a; Kobayashi et al. 2008; SCF 2002). It may be therefore stated that the analysed green coffee beans' place of geographical origin, as well as the local environmental pollution grade, causes significant differentiation in the PAHs contamination level for given coffee beans. Method of post-harvest coffee drying is also an important factor in this case.

Temperature, roasting method (flame-roasting, coal-grilling, gas roasting or electric oven-toasting) and its degree and raw products contamination level are, however, the main factors affecting PAHs level in coffee (Farah 2012; Houessou et al. 2007; Orecchio et al. 2009; Tfouni et al. 2013). Coffee roasting usually occurs at the temperature ranging from 185 to 220 °C in Polish food industry. As stated in the available literature concerning PAHs contamination, pyrolytic reactions occur during the above 170 °C roasting (Franca et al. 2005; Yeretzian et al. 2002). These are strongly linked with PAHs formation in food. Other studies revealed that Phen and Anthr formation in coffee occurs at above 220 °C temperature, whereas formation of Pyr, Chr, and B[a]A requires higher temperatures—from 250 to 260 °C (Farah 2012; Houessou et al. 2007). It is known that coffee beans which are roasted at high temperatures for a long time turn dark, numerous cracks appear on their surface and inner, rich in fat structure inside them becomes visible (Yeretzian et al. 2002). This favours the formation of lipophilic PAHs, especially heavy PAHs, and determines their high contents in final products. It may be stated on the basis of obtained results that the relatively low level of PAHs contamination in analysed coffee samples (especially roasted coffee beans) was the effect of mild roasting. These particular roasting conditions caused the reduction in total PAHs content in roasted coffee indeed. The reason for this phenomenon is light PAHs volatility. Conversely, it might be assumed that the use of higher roasting temperature may result in an increase in the level of PAHs contamination in roasted coffee beans samples.

## Conclusion

In order to examine the effect of mild roasting on the coffee beans' PAHs contamination, PAHs were determined in green Arabica and Robusta coffee beans and also after roasting using an electric coffee roaster. The obtained method performance parameters proved the suitability of the applied method in case of 19 PAHs analysis in coffee samples. The results of the study showed that the contamination level of roasted coffee beans was lower in comparison with that of their raw equivalents in the field of statistical significance. It was therefore proved that mild roasting conditions with the electrical heating system have a significant effect on PAHs contamination level in the final product and do not lead to the occurrence of PAHs formation conditions. This especially concerns heavy PAHs. This is the reason why mild roasting applied in the experiment may prove useful in case of reduction of PAHs contamination level, especially due to relatively high volatility of light PAHs. Moreover, relatively low level of PAHs content, in particular, the heavy ones, in analysed roasted Arabica and Robusta coffee beans may undoubtedly result in low contamination level of their infusions. However, considering that natural roasted coffee is a very popular beverage and the coffee beans roasting methods themselves vary considerably, further examination of PAHs contamination level in coffee is still required.

## References

[CR8] Chung SY, Yettella RR, Kim JS, Kwon K, Kim MC, Min DB (2011). Effects of grilling and roasting on the levels of polycyclic aromatic hydrocarbons in beef and pork. Food 
Chem.

[CR5] Ciecierska M, Obiedziński MW (2010). Polycyclic aromatic hydrocarbons in infant formulae, follow-on formulae and baby foods available in the Polish market. Food Control.

[CR6] Ciecierska M, Obiedziński MW (2013). Polycyclic aromatic hydrocarbons in the bakery chain. Food Chem.

[CR7] Ciecierska M, Obiedziński MW (2013). Polycyclic aromatic hydrocarbons in vegetable oils from unconventional sources. Food Control.

[CR2] Commission of the European Communities (2005) Commission Recommendation (EC) No. 108/2005 of 4 February 2005 on the further investigation into the levels of polycyclic aromatic hydrocarbons in certain foods. Off J Eur Union L 34/3

[CR1] Commission of the European Communities (2011). Commission Regulation (EU) No. 835/2011 of 19 August 2011 amending Regulation (EC) No. 1881/2006 as regards maximum levels for polycyclic aromatic hydrocarbons in foodstuffs. Off J Eur Union L.

[CR3] Commission of the European Communities (2011b) Commission Regulation (EU) No. 836/2011 of 19 August 2011 amending Regulation (EC) No. 333/2007 laying down the methods of sampling and analysis for the official control of the levels of lead, cadmium, mercury, inorganic tin, 3-MCPD and benzo(a)pyrene in foodstuffs. Off J Eur Union L 215/9

[CR9] European Food Safety Authority (EFSA) (2008). Polycyclic aromatic hydrocarbons in food—scientific opinion of the panel on contaminants in the food chain adopted on 9 June 2008. EFSA J.

[CR10] Farah A, Chu YF (2012). Coffee constituents. Emerging health effects and disease prevention.

[CR11] Franca AS, Mendonҫa JCF, Oliveira SD (2005). Composition of green and roasted coffees of different cup qualities. LWT Food Sci Technol.

[CR12] Guatemala-Morales GM, Beltrán-Medina EA, Murillo-Tovar MA, Ruiz-Palomino P, Corona-González RI, Arriola-Guevara E (2016). Validation of analytical conditions for determination of polycyclic aromatic hydrocarbons in roasted coffee by gas chromatography-mass spectrometry. Food Chem.

[CR15] Hong WJ, Jia H, Li YF, Sun Y, Wang L (2016). Polycyclic aromatic hydrocarbons (PAHs) and alkylated PAHs in the coastal seawater, surface sediment and oyster from Dalian, Northeast China. Ecotoxicol Environ Saf.

[CR13] Houessou JK, Delteil C, Camel V (2006). Investigation of sample treatment steps for the analysis of polycyclic aromatic hydrocarbons in ground coffee. J Agric Food Chem.

[CR16] Houessou JK, Maloug S, Leveque AS, Delteil C, Heyd B, Camel V (2007). Effect of roasting conditions on the polycyclic aromatic hydrocarbon content in ground Arabica coffee and coffee brew. J Agric Food Chem.

[CR14] Houessou JK, Goujot D, Heyd B, Camel V (2008). Modeling the formation of some polycyclic aromatic hydrocarbons during the roasting of Arabica coffee samples. J Agric Food Chem.

[CR17] Kobayashi R, Okamoto RA, Maddalena RL, Kado NY (2008). Polycyclic aromatic hydrocarbons in edible grain: a pilot study of agricultural crops as a human exposure pathway for environmental contaminants using wheat as a model crop. Environ Res.

[CR19] Lai JP, Niessner R, Knopp D (2004). Benz[a]pyrene imprinted polymers: synthesis, characterization and SPE application in water and coffee samples. Anal Chim Acta.

[CR18] Lee K, Shin H (2010). Determination of polycyclic aromatic hydrocarbons in commercial roasted coffee beans. Food Sci Biotechnol.

[CR20] Murkovic M, Pedreschi F, Ciesarova Z (2018). Process contaminants: a review. Ref Module Food Sci.

[CR21] Orecchio S, Ciotti VP, Culotta L (2009). Polycyclic aromatic hydrocarbons (PAHs) in coffee brew samples: analytical method by GC-MS, profile, levels and sources. Food Chem Toxicol.

[CR22] Pissinatti R, Nunes CM, Souza AG, Junqueira RG, Souza SVC (2015). Simultaneous analysis of 10 polycyclic aromatic hydrocarbons in roasted coffee by isotope dilution gas chromatography-mass spectrometry: optimization, in-house method validation and application to an exploratory study. Food Control.

[CR4] Scientific Committee on Food (SCF) (2002) Polycyclic aromatic hydrocarbons—occurrence in foods, dietary exposure and health effects. Report No. SCF/CS/CNTM/PAH/29 Add1 Final, 4 December 2002. https://ec.europa.eu/food/sites/food/files/safety/docs/sci-com_scf_out153_en.pdf. Accessed 15 April 2018

[CR24] Singh L, Varshney JG, Agarwal T (2016). Polycyclic aromatic hydrocarbons’ formation and occurrence in processed food. Food Chem.

[CR23] Stanciu G, Dobrinas S, Birghila S, Popescu M (2008) Determination of organic compounds from different types of coffee by HPLC and GC-ECD analysis. Environ Eng Manag J 7: 661–666. https://www.eemj.icpm.tuiasi.ro/pdfs/vol7/no6/EEMJ_Vol7_Nr6_2008.pdf#page=20. Accessed 2 May 2018

[CR25] Surma M, Sadowska-Rociek A, Cieślik E (2018). Assessment of thermal processing contaminant levels in dried and smoked fruits. Eur Food Res Technol.

[CR27] Tfouni SAV, Serrate CS, Leme FM, Camargo MCR, Teles CRA, Cipolli KMVAB, Furlani RPZ (2013). Polycyclic aromatic hydrocarbons in coffee brew: influence of roasting and brewing procedures in two Coffea cultivars. LWT Food Sci Technol.

[CR26] Tomaz S, Jaffrezo JL, Favez O, Perraudin E, Albinet A (2017). Sources and atmospheric chemistry of oxy- and nitro-PAHs in the ambient air of Grenoble (France). Atmos Environ.

[CR28] Yeretzian Ch, Jordan A, Badoud R (2002). From the green bean to the cup of coffee: investigating coffee roasting by on-line monitoring of volatiles. Eur Food Res Technol.

